# A review on phytochemical and pharmacological properties of *Litsea coreana*

**DOI:** 10.1080/13880209.2017.1302482

**Published:** 2017-03-17

**Authors:** Xuejing Jia, Peng Li, Jianbo Wan, Chengwei He

**Affiliations:** State Key Laboratory of Quality Research in Chinese Medicine, Institute of Chinese Medical Sciences, University of Macau, Macao, China

**Keywords:** *Litsea coreana*, phytochemistry, biological activity

## Abstract

**Context:** *Litsea coreana* H. Lév. (Lauraceae) is used as an ethnic herb or beverage in China. Substantial studies indicate that it contains a variety of compounds and shows diverse bioactivities with no toxicity.

**Objective:** This review analyzes and summarizes the ethnopharmacological applications, phytochemistry, and pharmacological activities and molecular mechanisms of *L. coreana*.

**Methods:** Related literature (from 1998 to 2016) was obtained and compiled via searching databases including Scopus, Web of Science, Google Scholar, CNKI and PubMed. Keywords (*Litsea coreana*, hawk tea, eagle tea and laoying cha) were used to select the articles.

**Results:** Studies indicate that *L. coreana* contains characteristic polysaccharides, polyphenols, essential oils, and numerious flavonoids, which exhibit remarkable bioactivities, such as hepatoprotection, hyperglycaemia, anti-inflammation, antioxidation and antibacterial, through multiple molecular mechanisms.

**Conclusion:** This paper provides a systematic review on the phytochemicals and pharmacological activities of *L. coreana* which should be useful for further study and application of this medicinal herb.

## Introduction

*Litsea coreana* H. Lév. (Lauraceae) has been used for tea production for hundreds of years in China. This tea is named hawk tea, eagle tea, or laoying cha in China, since the tree of *L. coreana* can be as high as 10 m and hawks usually rest and build nests on these trees (Xiang & Lu 1998). *L. coreana* was documented in a classical traditional Chinese medicine book ‘Ben Cao Gang Mu’ (Li Shizhen, Ming dynasty, AD 1590), and was used as a hypolipidemic herb in rural areas (Wang et al. 2009). *L. coreana* is widely distributed in many provinces of China, including Guizhou, Hubei, Anhui, Sichuan, Chongqing, Zhejiang, etc. The ethnomedical and drinking applications of hawk tea have been spread out in the district of Yangtze upriver and Guizhou province of China.

**Figure 1.**
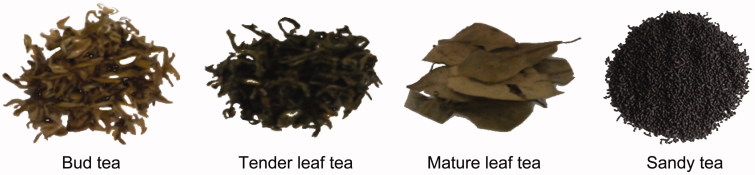
Four kinds of common hawk teas.

Hawk tea stems from the family Lauraceae and is different from green tea (Camelliaceae). According to the different degrees of maturity, hawk tea is divided into bud hawk tea (buds), hawk tea (tender and mature leaves) (Figure 1). Meanwhile, because of different processing methods, hawk tea has several types, *bai cha* (tender leaves are directly insolated or air dried), *dashu cha* (stems are directly sliced and decocted with water), and insect hawk tea or sandy tea (Figure 1), which is the feces of larvae of *Aglossa dimidiate* or *Hydrillodes morosa* that is fed with fresh mature leaves of *L. coreana* trees (Lu et al. 2001).

The leaves of *L. coreana* are abundant in proteins, amino acids, vitamins, sugars, polyphenols and flavonoids, but caffeine was not detectable (Ye & Yu 2001; Shu et al. 2013). Except for the above chemical constituents, the sandy tea is rich in fatty acids (Xu et al. 2000). Contents of trace elements, such as Pb, Cd, Mn, Fe, Zn, and Ca, in hawk teas from different areas are varied (Gu & Peng 2013). Its infusion has slight odor of camphor and aromaticity. Importantly, this folk beverage could prevent food spoilage, abdominal distension and sunstroke (Li & Zhang 2005).

It was found that collection time of *L. coreana* leaves could affect the chemical components (Han et al. 2014). The extracts of *L. coreana* leaves were analyzed by high-performance liquid chromatography equipped with photodiode array detector. The contents of kaempferol-3-*O*-β-d-glucoside and total flavonoids were as high as 8 and 31%, respectively (Ma et al. 2011). March is the optimal harvest month of hawk tea because of its high total polyphenol content and strong antioxidant activity (EC_50_ value of DPPH radical scavenging ability is 5.29 μg/mL) (Xiao et al. 2015). In this review, we focus on the latest research progress of the phytochemical components and pharmacological activity of *L. coreana*. More intensive studies are strongly expected to promote the development and application of *L. coreana* in food, beverage and pharmaceutical industry. Furthermore, this article provides systematic information and insights which could be interesting to researchers in related areas.

## Phytochemistry of *L. coreana*

Comprehensive research has been conducted to elucidate the structures of chemical constituents, such as flavonoids, polyphenols, essential oils and polysaccharides, from *L. coreana* using gas chromatography mass spectrometry (GC-MS), 1 D and 2 D nuclear magnetic resonance (NMR), and infrared spectrometry (IR). Among these compounds, flavonoids were considered to be the most dominant component (Ye & Yu 2004). Previous review articles have listed and classified some of the compounds in the genus *Litsea* (Agrawal et al. 2011; Kong et al. 2015; Wang et al. 2016). Here we specifically summarize the compounds extracted from *L. coreana*, one of the most popular and valuable species of *Litsea*.

## Chemicals of *L. coreana*

**Figure 2.**
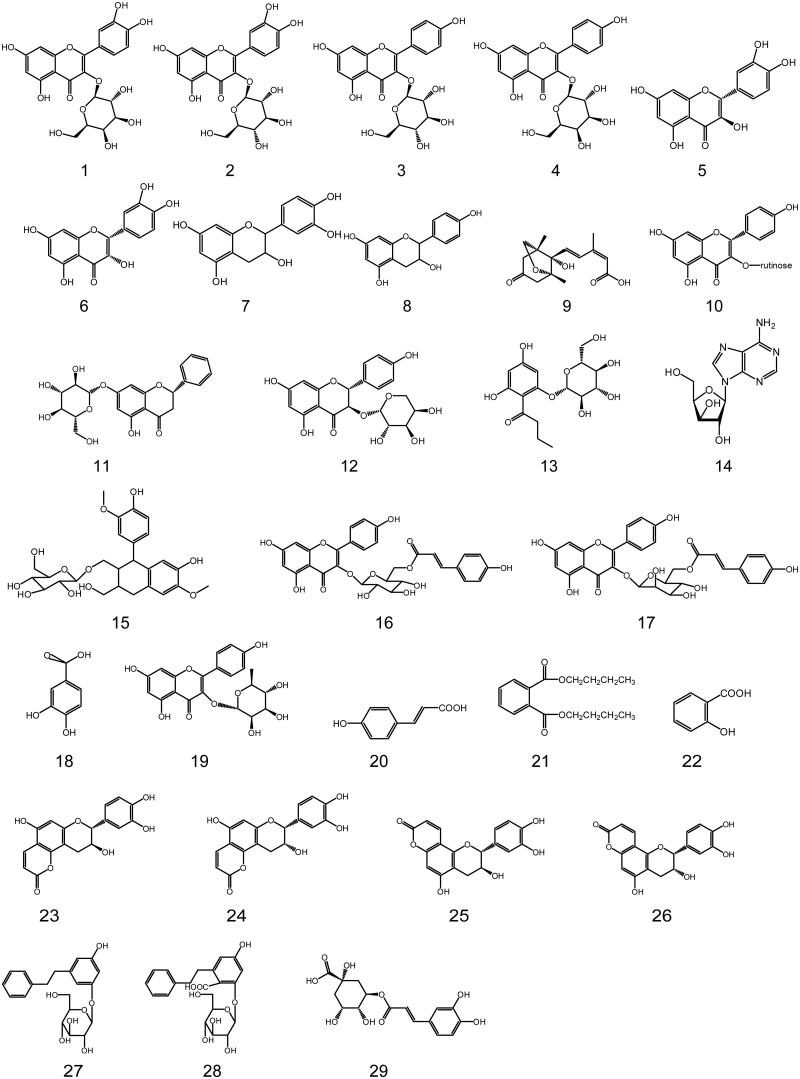
Structure of chemical components from *L. coreana.*

A number of recent high-profile reports indicated that bioactive phytochemicals of *L. coreana* had been isolated and reported. The major active constituents were flavonoids. There were 29 monomeric compounds had been isolated from the stems and leaves of *L. coreana* and their structures are presented in Figure 2.

Flavonoids of *L. coreana* were mainly composed of six monomers, including quercetin-3-*O*-β-d-galactopyranoside (**1**), quercetin-3-*O*-β-d-glucopyranoside (**2**), kaempferol-3-*O*-β-d-galactopyranoside (**3**), kaempferol-3-*O*-β-d-glucopyranoside (**4**), catechin (**5**), and epicatechin (**6**) (Chen et al. 2008).

Subsequent researchers isolated more compounds from the leaves of *L. coreana*, including quercetin (**7**), kaempferol (**8**), phaseic acid (**9**), kaempferol-3-*O*-β-d-rutinose (**10**), pinocembrin-7-*O*-β-d-glucopyranoside (**11**), aromadedrin-3-*O*-α-l-arabinopyranoside (**12**), 2,4,6-trihydroxybutyrophenone-2-*O*-β-d-glucopyranoside (**13**), adenoside (**14**), (+)-isolariciresinal-9-*O*-β-d-glucopyranoside (**15**), kaempferol-3-*O*-β-d-(6-*O*-trans-p-coumaroyl) glucopyranoside (**16**), kaempferol-3-*O*-β-d-(6-*O*-trans-p-coumaroyl) mannopyranoside (**17**), protocatechuic acid (**18**), kaempferol-3-α-l-rhamnose (**19**), trans-p-coumaric acid (**20**), n-butyl phthalate (**21**), salicylic acid (**22**), isophyllocoumarin (**23**), isoepiphyllocoumarin (**24**), phyllocoumarin (**25**), epiphyllocoumarin (**26**), 5-(2-phenylethyl)-3-hydroxyphenol-1-*O*-β-d-glucopyranoside (**27**), 6-(2-phenylethyl)-2,4-dihydroxy benzoic acid-2-*O*-β-d-glucopyranoside (**28**) and chlorogenic acid (**29**) (Yu et al. 2001b; Meng et al. 2012; Zhang et al. 2012b; Tang et al. 2013a, 2013b; Wang et al. 2014; Tan et al. 2016).

Aside from these monomeric compounds, other components from *L. coreana* were also reported. The essential oils of *L. coreana* leaves were determined, and the main compositions were decanal (71.53%), 10-undecenal (5.02%), *n*-nonaldehyde (3.95%), copaene (3.58%), dodecanoic acid, and ethenyl ester (2.63%) (Yu et al. 2001a). Furthermore, hawk tea contains vitamins and 17 amino acids (Ye & Yu 2001). Aqueous extracts of hawk bud tea, hawk primary leaf tea, and hawk mature leaf tea contain many bioactive constituents, such as polyphenols, flavonoids, vitamin C, and carbohydrates (Yuan et al. 2014). The protein hydrolysates of hawk teas contained 16 amino acids except for tryptophan (Jia et al. 2013a).

## Extraction technologies of *L. coreana*

Flavonoids played an important role in *L. coreana*. Thus, extensive studies focused on the extraction and isolation of flavonoids from *L. coreana.* The flavonoids were extracted by Soxhlet extraction method and determined using colorimetric method, and its yield was about 2.93% (Yang et al. 2011). After optimizing the extraction conditions, its yield was up to 6.19% (quercetin equivalents). The extraction parameters based on orthogonal experiment were: ethanol concentration, 70%, ratio of liquid to material, 35 mL/g, extraction temperature, 60 °C, extraction time, 90 min (Ji et al. 2011a).

Some ancillary methods have been used to increase the efficiency and yield of flavonoids. An optimum ultra-high pressure extraction method was used, and the optimized parameters were as followed: extraction pressure, 427 MPa, pressure holding time, 9 min, liquid-solid ratio, 41 mL/g, ethanol concentration, 70%, under such conditions, the yield of flavonoids was about 6.23% (Ji et al. 2011d). The microwave-assisted extraction conditions of mature leaf hawk tea were studied based on response surface methodology. The best extraction parameters were: microwave time, 61 s, microwave power, 560 W, ratio of water to solid, 10 mL/g, and ethanol concentration, 80.3%, under these conditions, the maximum extraction yield of flavonoids was around 6.52% (Jia et al. 2013c). The optimal cellulase extraction parameters were as follows: enzymatic hydrolysis temperature, 50 °C, pH of enzyme solution, 5.0, the time of enzymatic hydrolysis, 120 min, and the concentration of cellulase, 0.4 mg/mL. Under these conditions, the content of flavonoids was 2.68% (Yang 2011). Compared with the method of ethanol-*n*-butanol extraction, the water-macroporous resin extraction method allows to extract higher content of total flavonoids from *L. coreana* leaves, which was suitable for industrial production (Lu et al. 2009).

The extraction conditions of total saponins were also optimized. The optimum ultrasound extraction conditions were obtained as follows: ultrasound power, 450 W, extraction time, 40 min, extraction temperature, 60 °C, and solvent-sample ratio, 30 mL/g. Under above conditions, the yield of total saponins was 75.4 mg/g (Wang et al. 2012b). In addition, the optimum microwave-assisted extraction conditions were as follows: liquid to material ratio, 40 mL/g, microwave power, 480 W, microwave time, 60 s, and ethanol volume, 60%, under these conditions, the yield of total saponins was about 71.6 mg/g (ginsenoside Rg1 equivalents) (Zhang et al. 2012a).

Furthermore, some other components were isolated, such as polyphenols, polysaccharides, and aqueous extracts. The polyphenolic compounds of eagle tea were purified by polyamide chromatography, and the yield was about 18.06% (Shen et al. 2010). Hawk mature leaf tea had the advantage of low price and rich natural resources, thus, the extraction conditions of its polysaccharides were optimized. The best extraction parameters were: extraction temperature, 88.9 °C, extraction time, 128.2 min, and ratio of water to solid, 11.4 mL/g, the maximum yield was about 12.74% (Jia et al. 2014). The optimal ultrasound-assisted extraction conditions of aqueous extracts were: ultrasound power, 200 W, ultrasound temperature, 93 °C, and ultrasound time, 16 min. Using these conditions, the yield of aqueous extracts from *L. coreana* was 38.84% (Li et al. 2013).

## Pharmacological activities of *L. coreana*

### Hepatoprotective activity

Rat models of liver injury were used to investigate the hepatoprotective activity of *L. coreana*. Methanol extracts of hawk tea had preventive effects against hepatic damage. In carbon tetrachloride-induced Sprague–Dawley rat hepatic damage model, treatment with 400 mg/kg methanol extracts reduced the serum levels of proinflammatory cytokines, such as interleukin-6 (IL-6), interferon-γ (IFN-γ) and tumour necrosis factor-α (TNF-α). Moreover, methanol extracts decreased mRNA and protein expressions of inflammation-related genes in liver, including inducible nitric oxide synthase (iNOS), cyclooxygenase-2 (COX-2), TNF-α and IL-1β (Zhao 2013). Kaempferol-glucopyranoside could inhibit the proliferation of rat hepatic stellate cells, the major cell type involved in liver fibrosis in response to liver damage, via down-regulating the mRNA expressions of collagen I, collagen III, Smad2, Smad3, and up-regulating the mRNA expression of Smad7 (Zhou et al. 2010b).

Total flavonoids of *L. coreana* (TFLC) showed a remarkable protective effect on liver damage. In a nonalcoholic steatohepatitis rat model, TFLC (200 mg/kg) significantly reduced the triglycerides and malondialdehyde (MDA) levels of serum and liver tissue, and increased the activities of superoxide dismutase (SOD) of liver tissue (Ni et al. 2006). Additionally, treatment with TFLC 200 mg/kg reduced the serum levels of alanine aminotransferase (ALT), aspartate aminotransferase (AST), triglycerides, total cholesterol and TNF-α, and decreased the levels of triglycerides, total cholesterol and the excess lipids accumulation in liver. Furthermore, TFLC suppressed the mRNA expression of TLR4 and protein expression of NF-κB (Wang et al. 2012a). Moreover, TFLC showed a protective effect on alcoholic fatty liver in rats. In an alcoholic fatty liver rat model, treatment with 200 mg/kg TFLC reduced the serum levels of triglycerides, total cholesterol, low-density lipoprotein (LDL)-cholesterol, TNF-α, glucose and insulin, and down-regulated the expression of hepatic adipose differentiation-related protein (Hu et al. 2012). TFLC (100 mg/L) also reduced the activities of ALT and AST and the level of triglycerides in steatotic hepatocytes, and decreased the mRNA expressions of adipose differentiation-related protein and peroxisome proliferator-activated receptor γ (PPARγ) (Hu et al. 2013).

In liver fibrosis rat model, TFLC (200 mg/kg) was able to ameliorate liver injury by reducing serum contents of ALT, AST, hyaluronic acid, laminin, procollagen III N-terminal peptide, procollagenase IV and hydroxyproline, and suppressing the expressions of α-smooth muscle actin, collagen I, transforming growth factor-β1 (TGF-β1) and transforming growth factor β receptor 1 (TGF-βR1) (Huang et al. 2010). In CCl_4_ induced liver fibrosis rat model, treatment with TFLC 200 mg/kg reduced the serum levels of ALT, AST, hyaluronic acid, laminin, procollagen III N-terminal peptide, procollagenase IV, collagen I, leptin, and TGF-β1. Additionally, TFLC suppressed the mRNA and protein expressions of leptin receptor (Ob-Rb), TGF-βR1, and Smad3 in liver (Huang et al. 2012).

For high fat diet-induced hepatic steatosis rat model, treatment with TFLC (200 mg/kg) for 4 weeks greatly reduced the levels of ALT, AST, lipids accumulation and TNF-α in serum. Simultaneously, TFLC could increase the levels of leptin and insulin in serum, increase the levels of peroxisome proliferator-activated receptor α (PPARα), SOD and MDA, and decrease the accumulation of lipids in liver (Wang et al. 2009).

Besides, TFLC could improve insulin resistance. In hyperlipidemia and insulin resistance rat model, TFLC (200 mg/kg) improved the state of impaired glucose tolerance. TFLC significantly depressed the serum levels of fasting serum glucose and insulin, total cholesterol, triglycerides, low-density lipoprotein cholesterol, free fatty acids and leptin, and obviously increased the content of high-density lipoprotein cholesterol and index of insulin sensitivity (Lv et al. 2009).

### Hypoglycaemic activity

TFLC from *L. coreana* is reported to reduce blood glucose and relieve hyperglycaemia. In a streptozocin-induced type 2 diabetic rat model, orally administered with TFLC (400 mg/kg) increased the contents of high-density lipoprotein (HDL) cholesterol and SOD, and decreased the body weight and the serum levels of free fatty acids, total cholesterol, triglycerides, LDL cholesterol, C-reactive protein and MDA. In addition, it downregulated the expression of protein tyrosine phosphatase 1B in liver (Lu et al. 2010).

In addition, in the type 2 diabetic rat model, treatment with TFLC 400 mg/kg reduced the levels of fast blood glucose, glycohemoglobin, fast blood insulin, free fatty acids, total cholesterol, triglycerides and LDL-cholesterol in serum, and enhanced the content of MDA in liver. Furthermore, TFLC increased the levels of HDL cholesterol in serum and SOD activity in liver (Sun et al. 2010).

### Anti-inflammatory activity

Many studies have shown that TFLC could interrupt the process of inflammation. In a complete Freund's adjuvant-induced arthritis (AA) rat model, administration of TFLC (50 mg/kg) significantly suppressed the primary and secondary paw swelling, Concanavalin A or lipopolysaccharides (LPS)-induced splenocyte proliferation, and pathological damage of knee joint. TFLC reduced the levels of IL-1, TNF-α and IL-6 in peritoneal macrophages and inhibited the expression of protein matrix metalloproteinases (MMP-9). Meanwhile, TFLC promoted IL-2 production of splenocytes. Together, TFLC had a therapeutic effect on AA rats via decreasing the production of inflammatory cytokines and inhibiting the expression of MMP-9 (Wang et al. 2007, 2008). Another study on AA demonstrated that TFLC (100 mg/kg) diminished secondary paw swelling and suppressed serum levels of TNF-α and IL-1β, and reduced the expression of mTOR complex 1 and TNF-α in peritoneal macrophages (Zhong et al. 2014). In collagen II-induced arthritis rat model, TFLC (50 mg/kg) inhibited the paw swelling and increased body weight of rats. In addition, TFLC (0.05 mg/L) reduced the level of IL-2 in splenocytes and the mRNA expression of TNF-α, IL-1 and TNF-α in peritoneal macrophage (Zhou et al. 2010a). In LPS-activated primary mouse peritoneal macrophage model, compounds **23**–**26** could inhibit the production of TNF-α and IL-1 (Tang et al. 2013b). In LPS-induced RAW 264.7 cell model, compounds **27**–**28** could inhibit the production of TNF-α and IL-1 (Tang et al. 2013a), indicating that these compounds from *L. coreana* could significantly inhibit inflammatory responses.

### Antioxidant activity

The extracts and fractions from *L. coreana* show potent antioxidant abilities *in vitro*. It was reported that the ethanol extracts of hawk tea exhibited strong antioxidant activities against 2,2′-azino-bis (3-ethylbenzothiazoline-6-sulphonic acid) (ABTS) radical, 2,2-diphenyl-1-picrylhydrazyl (DPPH) radical, and hydroxyl radical, with EC_50_ values (concentration for 50% of maximal effect) of 1.09, 0.06, 2.42 mg/mL, respectively. Moreover, the extracts had strong inhibitory effects on linoleic acid peroxidation, with an inhibition rate of 65.5% (Tan et al. 2016). The purified components, TFLC, had strong DPPH radical scavenging ability and reducing power, with EC_50_ values of 18.17 and 77.52 g/L, respectively (Ji et al. 2011a). Eight flavonoid monomers, hyperin, isoquercitrin, quercitrin, quercetin, kaempferol, catechins, chlorogenic acid and epicatechin showed ABTS radical scavenging activities (Meng et al. 2012).

Total saponins also showed strong DPPH radical scavenging activity and relative reducing ability. At 160 μg/mL, its scavenging rate was 93.53%, which was better than that of butylated hydroxytoluene (Zhang et al. 2012a). More importantly, the aqueous extracts of bud hawk tea and hawk tea of tender and mature leaves demonstrated strong DPPH radical scavenging activity and ferric reducing activity power, prevented erythrocyte haemolysis, and avoided formation of LDL conjugated diene (Yuan et al. 2014).

Other active constituents, like peptides and polysaccharides, revealed strong antioxidant activities. The protein hydrolysates showed strong DPPH radical scavenging activities and iron chelating activity, as well as potent solubility and emulsifying properties (Jia et al. 2013a). Three crude polysaccharides were separately isolated from bud hawk tea and hawk tea of tender and mature leaves. These polysaccharides had various physicochemical properties and showed obvious antioxidant activities against DPPH radical, ferric oxidation, hydroxyl radical and erythrocyte hemolysis (Jia et al. 2013b). The polysaccharides of hawk tea of mature leaves were purified by chromatography of DEAE-52, and its fractions, encompassed arabinose, galactose, glucose and mannose, showed better antioxidant activities against DPPH radical and ferric oxidation than that of crude polysaccharides (Jia et al. 2014).

### Antimicrobial activity

Ethanol extracts of *L. coreana* leaves promoted the growth of the anaerobes, e.g. *Lactobacillus* and *Bifidobacterium*, in the depth of the mice intestinal canals (Wu et al. 2012). TFLC (40 mg/mL) had significant inhibitory effects on *Bacillus anthraci*, *Proteusbacillus vulgaris*, *Staphyloccocus aureus*, and *Bacillus subtilis* (Ji et al. 2011c). Total saponins of *L. coreana* (25 mg/mL) could also potently inhibit the growth of *Enterobacter aerogenes*, *Proteusbacillus vulgaris* and *Staphylococcus aureus* (Wang et al. 2012b). In addition, aqueous extracts of *L. coreana* leaves could regulate the numbers of aerobic bacterium, enterobacterium and enterococcus, which existed on the surface of the mouse intestinal canals (Wu et al. 2012).

### Other pharmacological activities

The methanol extracts of *L. coreana* leaves had a conspicuous anti-HSV-1 activity with an IC_50_ value of 12.02 μg/mL. The extracts could directly inactivate virus and stop the attachment of virus to Vero cells (Xu et al. 2011). The ethanol extracts of *L. coreana* leaves (400 μg/mL) showed inhibitory effects on the growth of human gastric carcinoma AGS cells and colon carcinoma HT-29 cells, and the ethanol extracts (1.25 mg) had anti-mutagenic properties determined by the Ames test (Zhao et al. 2008). Epigallocatechin gallate of *L. coreana* might be connected with α-glucosidase inhibitory activity with an IC_50_ of 3.8 mg/mL, which was better than that of radix glycyrrhizae extracts (Li et al. 2010).

When TFLC (200 mg/kg) was intraperitoneally injected to cyclophosphamide-treated mice, it notably increased carbon clearance indexes and phagocytic values of macrophages, and obviously augmented the levels of immunoglobulin M (IgM) and immunoglobulin G (IgG) in serum, the content of hemolysin in splenocytes, the ratio of CD4^+^ and CD8^+^ T cells, and IL-2 production (Hu et al. 2007). TFLC (100 mg/mL) increased the cytotoxicity of oxaliplatin in mouse testicular cancer I-10 cells via gap junction-mediated regulation and enhanced the induction of tumour cell apoptosis by up-regulation of Bax/Bcl-2 ratio and caspase-3/9 expression (Yu et al. 2014a). Furthermore, TFLC (20 μg/mL) enhanced the gap junction intercellular communication (GJIC) of mouse TM3 testicular Leydig cells. TFLC increased the expression of total Connexin 43 (Cx43) protein and the membrane Cx43 protein, which was responsible for the enhanced GJIC and synergistic anticancer effect (Yu et al. 2014b). In a focal cerebral ischaemia/reperfusion injury rat model, TFLC (100 mg/kg) alleviated cerebral ischemia-induced neurological deficits. In detail, TFLC reduced the levels of nitrates plus nitrites, MDA and lactate dehydrogenase, and increased the levels of glutathione, SOD and catalase (Dong et al. 2013).

The aqueous extracts of hawk tea could defend fibroblasts against UV irradiation, especially at relatively low concentrations (2.5 and 1.25 mg/mL). Its protective effect was better than green tea aqueous extracts (Chen et al. 2005).

### Toxicity

There are only few papers so far to demonstrate the toxicity of *L. coreana*, which focused on the total flavonoids. When pretreated with TFLC (100 mg/L) for 48 h, it had no obvious effect on the survival of normal hepatocytes (Hu et al. 2013). The mean body weight and behavioural changes of normal rats were observed for 14 days when the rats were orally treated with TFLC (10 g/kg). The results indicated that there was no detectable sign of toxic responses in the tested rats, suggesting that TFLC was nontoxic and safe (Wang et al. 2008). Concerning the limited toxicity data, more studies are needed to evaluate the acute and chronic toxicity of *L. coreana* extracts and components in different animal models.

### Application and limitation

Currently, there have been a wide planting area and rich natural resources of *L. coreana*, which are continuously processed into tea or medicinal herbs. Researchers have made significant progress in isolating chemical compounds and evaluating their biological activities. However, there is still much potential work to improve the utilization and development of *L. coreana*. (1) More components, except for total flavonoids, from *L. coreana* need to be investigated for their bioactivities and underlying molecular mechanisms. (2) Pre-clinical and clinical studies are needed to assess the safety and efficacy of components in *L. coreana*. Particularly, TFLC, a mixture of 6 monomeric flavonoids, shows remarkable hepatoprotective and anti-inflammatory activities. TFLC has the potential to be developed as a promising therapeutic medicine, like Ginkgo Biloba flavonoids, also a mixture of compounds, which exhibit potent beneficial effects on hypertension, aging, dementia and Alzheimer’s disease (Smith & Luo 2004). (3) *Litsea coreana* could be utilized in functional food industry. Hawk tea extracts have diverse health benefits, thus, we can develop functional drinks or foods. For example, *L. coreana* powder can be used to brew beverage with jujube. This functional drink has a rich flavour and an intense sweet taste (Ji et al. 2011b). In addition, *L. coreana* powder can be used to make yogurt, since the extracts from *L. coreana* leaves can increase the numbers of lactic acid bacteria, *Streptococcus thermophilus*, *Lactobacillus acidophilus*, and *Lactobacillus casei* (Ye et al. 2012). Natural pigment from *L. coreana* leaves can be used as food color without toxic concerns. In addition, this pigment displays different colors in the solution with various pH values and metal ions, and it is stable for temperature variations (Ye & Yu 2002).

However, there are some shortcomings in the applications of *L. coreana* extracts. The pharmacological application of flavonoids could be affected by their poor absorption. Flavonoids of *L. coreana* could be transported by sodium-dependent glucose transporter. In a study using Madin Darby canine kidney cell monolayer model, the flavonoid glucosides of *L. coreana*, quercetin-3-*O*-β-d-glucoside and kaempferol-3-*O*-β-d-glucoside, were uptaken and transported by sodium-dependent glucose transporter. It was noted that multidrug resistance associated protein 2 could limit the absorption of these flavonoid glucosides (Chen et al. 2014).

Collectively, more attention and effort should be given to the investigation of phytochemical properties, nutritional and pharmacological activities of *L. coreana*, which could largely promote the development and application of this valuable resource in foods, beverages and pharmaceutical industries.

## Conclusion

As reviewed herein, studies revealed that *L. coreana* contained abundant flavonoid compounds, which demonstrated significant pharmacological activities, particularly hepatoprotective, anti-inflammatory and antioxidant activities. However, more studies are needed in the investigation of the responsible phytochemicals for their various pharmacological activities, which could dramatically enhance the research and development of *L. coreana*.
